# Expression and regulation of prostate androgen regulated transcript-1 (PART-1) and identification of differential expression in prostatic cancer

**DOI:** 10.1054/bjoc.2001.1883

**Published:** 2001-08

**Authors:** M Sidiropoulos, A Chang, K Jung, E P Diamandis

**Affiliations:** Department of Pathology and Laboratory Medicine, Mount Sinai Hospital, Toronto, Ontario, M5G 1X5, Canada; Department of Laboratory Medicine and Pathobiology, University of Toronto, Ontario, M5G 1L5, Canada; Department of Urology, University Hospital Charite, Humboldt University, Berlin, D-10098, Germany

**Keywords:** prostate cancer, androgen regulated transcript, quantitative RT-PCR, differentially expressed genes, tumour markers, PART-1

## Abstract

Prostate androgen regulated transcript 1 (PART-1), is a gene predominantly expressed in the prostate gland and is regulated by androgens in human prostate cancer cell lines. Here, we report additional characteristics of PART-1 tissue expression and hormonal regulation and study its expression profile in human normal and matched prostate cancer tissues. Since PART-1 shows similarity to prostate-specific antigen (PSA) in prostate specificity and regulation, we hypothesized that it may be implicated in prostate carcinogenesis or may be a potential new biomarker. We used reverse transcriptase polymerase chain reaction (RT-PCR) to further characterize PART-1 tissue expression and hormonal regulation in the LNCaP prostate cancer cell line. RT-PCR analysis revealed that PART-1 is expressed not only in the prostate and salivary gland, but also in other tissues, including the thymus and placenta. In addition to androgen stimulation, PART-1 is also up-regulated by progestins, oestrogens and glucocorticoids. We further studied the expression of PART-1 in 27 paired (from the same patient) cancerous and non-cancerous prostatic tissues, with qualitative and quantitative RT-PCR (LightCycler®technology), in order to examine whether PART-1 is overexpressed or underexpressed in cancer. Our results indicated that PART-1 is more frequently overexpressed in the cancerous prostatic tissue. We conclude that this gene is overexpressed in prostate cancer and may represent a novel prostate cancer tumour marker. © 2001 Cancer Research Campaign http://www.bjcancer.com


*British Journal of Cancer* (2001) **85**(3), 393–397
© 2001 Cancer Research Campaign
doi: 10.1054/ bjoc.2001.1883 available online at http://www.idealibrary.com on IDEAL®

http://www.bjcancer.com

### Expression and regulation of prostate androgen regulated transcript-1 (PART-1) and identification of differential expression in prostatic cancer

**M Sidiropoulos[1], A Chang[1,2], K Jung[3] and EP Diamandis[1,2]**

[1]Department of Pathology and Laboratory Medicine, Mount Sinai Hospital, Toronto, Ontario, M5G 1X5 Canada; [2]Department of Laboratory Medicine and Pathobiology, University of Toronto, Ontario, M5G 1L5 Canada; [3]Department of Urology, University Hospital Charite, Humboldt University, Berlin, D-10098 Germany

**Summary** Prostate androgen regulated transcript 1 (PART-1), is a gene predominantly expressed in the prostate gland and is regulated by androgens in human prostate cancer cell lines. Here, we report additional characteristics of PART-1 tissue expression and hormonal regulation and study its expression profile in human normal and matched prostate cancer tissues. Since PART-1 shows similarity to prostate-specific antigen (PSA) in prostate specificity and regulation, we hypothesized that it may be implicated in prostate carcinogenesis or may be a potential new biomarker. We used reverse transcriptase polymerase chain reaction (RT-PCR) to further characterize PART-1 tissue expression and hormonal regulation in the LNCaP prostate cancer cell line. RT-PCR analysis revealed that PART-1 is expressed not only in the prostate and salivary gland, but also in other tissues, including the thymus and placenta. In addition to androgen stimulation, PART-1 is also up-regulated by progestins, oestrogens and glucocorticoids. We further studied the expression of PART-1 in 27 paired (from the same patient) cancerous and non-cancerous prostatic tissues, with qualitative and quantitative RT-PCR (LightCycler® technology), in order to examine whether PART-1 is overexpressed or underexpressed in cancer. Our results indicated that PART-1 is more frequently overexpressed in the cancerous prostatic tissue. We conclude that this gene is overexpressed in prostate cancer and may represent a novel prostate cancer tumour marker. © 2001 Cancer Research Campaign http://www.bjcancer.com

**Keywords**: prostate cancer; androgen regulated transcript; quantitative RT-PCR; differentially expressed genes; tumour markers; PART-1

Prostate cancer is the second leading cause of death in males in the United States and is the most frequently diagnosed malignancy in men (Rhim, 2000). Early diagnosis of prostate cancer contributes in improving these detrimental statistics. Prostate cancer screening has provided a method for early prostate cancer detection and administration of curative treatments. Since screening appears to save lives, there is now a need for developing more biomarkers to improve efficiency (McDavid et al, 2000; Moran et al, 2000; Webb and Holmes, 2000). Prostate-specific antigen (PSA) analysis is widely used for prostate cancer monitoring, as well as for early detection (Oesterling, 1991; McCormack et al, 1995). PSA, which has prostate-specific expression, has been suggested to be implicated in the initiation and progression of prostate cancer (Diamandis, 2000). PSA levels in serum are frequently raised in benign prostatic hyperplasia (BPH), which is a major limitation. Development of a variety of techniques, which include age-specific reference ranges, PSA density, PSA velocity and percentage of free PSA have improved the clinical value of PSA (Oesterling, 1991; McCormack et al, 1995).

Many recent studies aim at identifying new genes that might have the potential to serve as diagnostic and/or prognostic markers for cancer. Human glandular kallikrein 2 is a serine protease produced by the prostate which is now used as an additional biomarker for the diagnosis and management of prostate cancer (Rittenhouse et al, 1998; Magklara et al, 1999; Heese et al, 2000).

PSA and hK2 were thought to be strictly expressed in the prostate, however, new studies have indicated that both kallikreins are expressed in diverse tissues and can be easily detected in many fluids (Black and Diamandis, 2000; Diamandis, 2000). PSA is produced in a high proportion of breast tumours and is a favourable indicator of prognosis (Black and Diamandis, 2000).

Our objective is to identify genes that may have the potential to be used as cancer markers. PART-1 (prostate androgen regulated transcript 1) is predominately expressed in the prostate and is transcriptionally-regulated by androgens in human prostate cancer cells (Lin et al, 2000). In this report we characterize PART-1 tissue expression and hormonal regulation by qualitative and quantitative RT-PCR and found that this gene is frequently up-regulated in prostate cancer tissues.

*Received 11 January 2001*
*Revised 27 March 2001*
*Accepted 5 April 2001*

*Correspondence to:* EP Diamandis

### MATERIALS AND METHODS

### Tissue expression

Total RNA isolated from 27 different human tissues was purchased from Clontech, Palo Alto, CA. Tissue cDNA was prepared and PCR amplified at various dilutions with three different sets of primers (Table 1). One primer set amplified PART-1, another the PSA transcript and the third set amplified the actin transcript (the last 2 transcripts served as controls).

**Table 1** Primers used for reverse RT-PCR analysis

| Gene | Primer name | Sequence |
|---|---|---|
| PART-1 | F1 | AAGGCCGTGTCAGAACTCAA |
| | R1 | GTTTTCCATCTCAGCCTGGA |
| PSA | PSAS | TGCGCAAGTTCACCCTCA |
| | PSAAS | CCCTCTCCTTACTTCATCC |
| Actin | ACTINS | ACAATGAGCTGCGTGTGGCT |
| | ACTINAS | TCTCCTTAATGTCACGCACGA |

All nucleotide sequences are given in the 5′ to 3′ orientation.

### Prostate cancer cell line and hormonal stimulation procedures

The prostate cancer cell line LNCaP was purchased from the American Type Culture Collection (ATCC), Rockville, MD. Cells were cultured in RPMI media (Life Technologies, Inc.) and supplemented with glutamine (200 mmol $l^{-1}$), bovine insulin (10 mg $l^{-1}$), fetal bovine serum (10%), antibiotics and antimycotics in plastic flasks, to near confluency. The cells were then aliquoted into 24-well tissue culture plates and cultured to 50% confluency. 24 h prior to each procedure, the culture media were changed into phenol red-free media containing 10% charcoal-stripped fetal bovine serum. For stimulation, various steroid hormones dissolved in 100% ethanol were added into the culture media, at a final concentration of $10^{-8}$ M. Cells stimulated with 100% ethanol were included as controls. The cells were grown for 24 h, after which they were harvested and total RNA extracted (see below).

### Reverse transcriptase polymerase chain reaction

Using Trizol™ reagent (Life Technologies, Inc) and following the manufacturer's instructions, we extracted total RNA from the cell line LNCaP. Through spectrophotometry, RNA concentrations were determined. Using the Superscript™ preamplification system (Life Technologies, Inc), 2 μg of total RNA was reverse-transcribed into first strand DNA. The gene-specific primers (Table 1) were designed through information on the genomic and cDNA structure of PART-1, obtained from NCBI. PCR reactions were carried out in a reaction mixture, containing 1 μl of cDNA, 1.5 mM $MgCl_2$, 2.5 μl of 25 mM PCR buffer, 0.5 μl of 10 mM dNTPs (deoxynucleoside triphosphates), 0.5 μl (150 ng) of primers, 20 μl of $H_2O$ and 0.25 μl (1 unit) of HotStar Taq polymerase (Qiagen, Valencia, CA) on an Eppendorf Mastercycler gradient system (Eppendorf, Westbury, NY). The cycling conditions were 95°C for 15 minutes to activate the HotStar Taq polymerase, followed by 35 cycles of 94°C for 30 s, 62°C for 1 minute and a final extension at 72 °C for 10 minutes.

Quantitative PCR was performed on a Roche LightCycler® system (Roche Molecular Biochemicals, Mannheim, Germany). PCR reactions were carried out in a reaction mixture consisting of 15.2 μl of $H_2O$, 2 mM $MgCl_2$, 0.5 μl (150 ng) of primers, 1 μl of cDNA and 2 μl of LightCycler DNA Master SYBR® Green I (Roche). Protocol conditions consisted of denaturation at 95°C for 10 minutes, followed by 40 cycles of 63°C for 55 s and 72°C for 40 s of extension and data acquisition, with a subsequent melting at 95°C for 1 s. Quantitative data analysis was made possible, through the use of PART-1 RNA from serially diluted prostate cDNA, using 1, 10, 100 and 1000-fold dilutions. These 4 samples provided the template on which a line of best fit was plotted and used as a standard curve for data interpretation after each run. Construction of standard curves and the tabulation of second derivative peaks displayed the beginning and end of the log-linear phase of PCR products.

The amplified PCR products were also separated using 1.5% agarose gels. The isolated bands were then purified using the Qiagen gel purification kit. The PCR products were cloned into the pCR 2.1-TOPO vector (Invitrogen, Carlsbad, CA, USA) in order to verify their identity by sequencing. The inserts were sequenced in both directions using vector-specific primers with an automated DNA sequencer.

### Screening for PART-1 transcripts in prostatic tissues

Included in this study were tissue samples from 27 patients with prostatic cancer, that had been surgically removed by radical retropubic prostatectomy. Patient age ranged from 50–68 years, with a median of 64. Cancerous and non-cancerous pieces of the whole prostates were carefully excised and verified by histopathological examination. All patients had a histologically confirmed diagnosis of primary cancer and received no treatment before surgery (except patient 1080, who was treated with antihormonal therapy 4 weeks prior to surgery).

All tissue samples were minced with a scalpel, on ice and immediately transferred into a 2 ml polypropylene tube. Tissue samples were then homogenized and RNA extracted using Trizol™ reagent (Life Technologies, Inc) following the manufacturer's recommendations. The RNA concentration was determined spectrophotometrically and 2 μg of total RNA was reverse-transcribed into first strand cDNA as described above.

### RESULTS

### Tissue expression of the PART-1 gene

As shown in Figure 1, the PART-1 gene is highly expressed in the prostate, placenta, thymus, and salivary gland, and at lower levels in trachea, kidney and brain. The PCR-products were subsequently sequenced to verify the RT-PCR specificity.

### Hormonal regulation of the PART-1 gene

To verify whether the PART-1 gene is under steroid hormone regulation, the prostate carcinoma cell line LNCaP was used. PSA, which is up-regulated by androgens and progestins, was used as a control gene. Our results show that PART-1 is up-regulated by dihydrotestosterone (DHT) and to a lower extent by oestradiol, norgestrel (a synthetic progestin) aldosterone and dexamethasone (Figure 2).

### Malignant versus non-malignant prostatic tissues

To determine the differential expression levels of PART-1 in normal (benign) and malignant (cancer) tissues, we analysed 27 pairs of prostatic tissue extracts (normal/cancer) through qualitative and quantitative PCR. We determined that 18 out of 27 patients had elevated PART-1 in the cancerous tissue, 7 had decreased PART-1 levels in the cancerous tissue compared to the non-cancerous tissue, while two patients had similar levels in both (Table 2). The differential expression of PART-1 between normal and tumour tissues was 93%, with 67% (18 out of 27) of the matched prostate samples displaying tumour-associated over

*© 2001 Cancer Research Campaign*

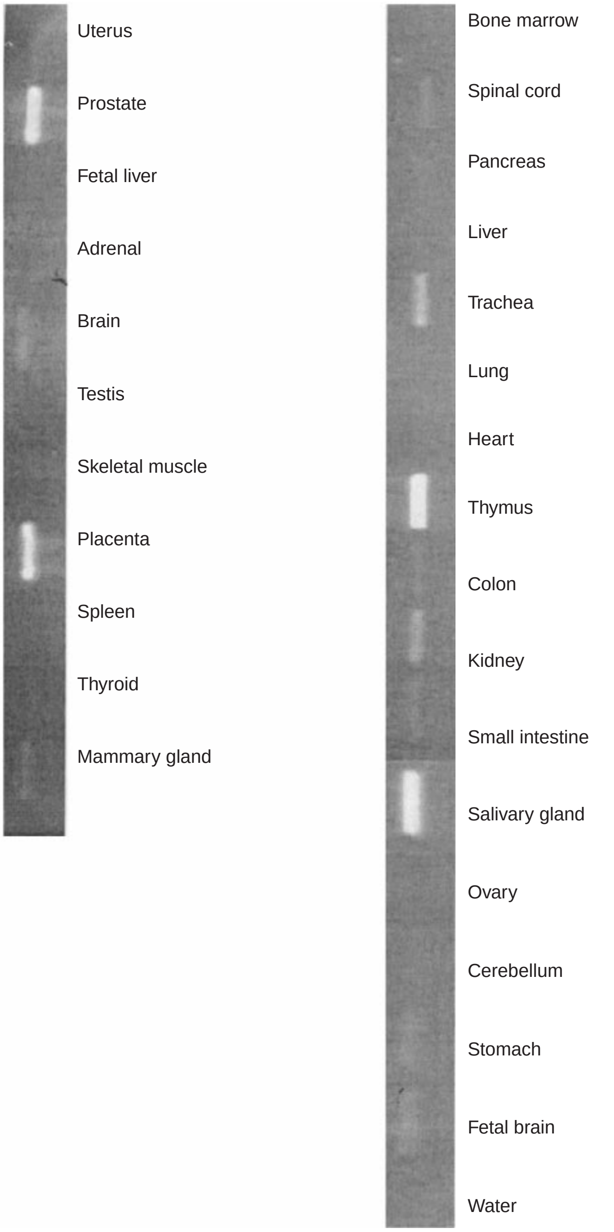

**Figure 1** Tissue expression of the PART-1 gene as determined by RT-PCR. PART-1 is highly expressed in prostate, placenta, thymus and salivary gland and to a lower extent in the trachea, kidney and brain and other tissues

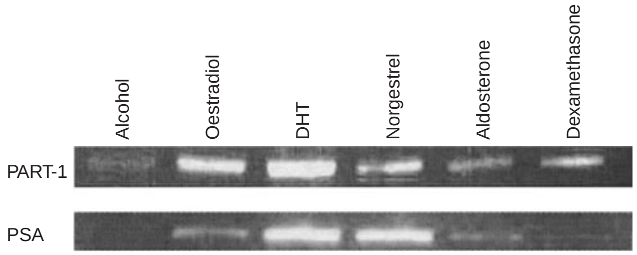

**Figure 2** Hormonal regulation of PART-1 gene in the LNCaP prostate carcinoma cell line. PSA, acting as a control gene is mainly up-regulated by dihydrotestosterone (DHT) and by the synthetic androgenic progestin norgestrel. PART-1 is mainly up-regulated by DHT, but also by oestradiol, norgestrel, aldosterone and dexamethasone

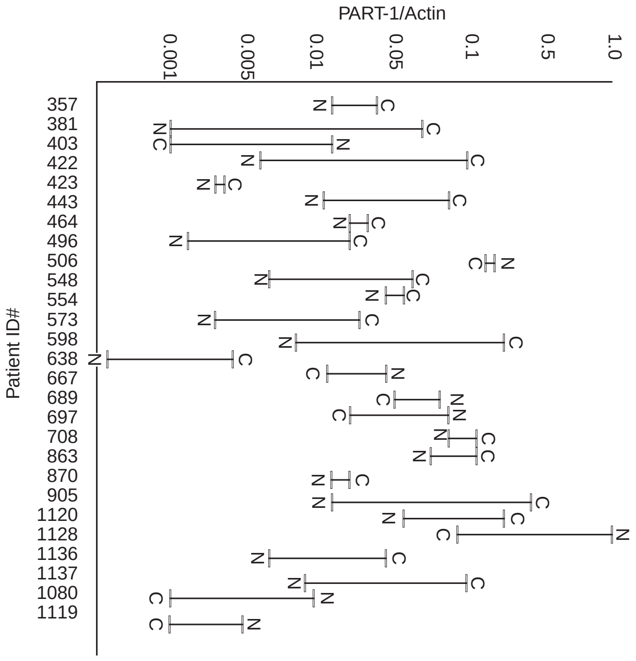

**Figure 3** Distribution of paired values for the expression of PART-1 in noncancerous (N) and cancerous(C) prostatic tissues for the 27 patients. The signal is expressed as a normalized ratio between PART-1 and actin gene. 18/27 patients demonstrate PART-1 upregulation in the cancerous tissue expression of PART-1. The distribution of quantitative data in the cancerous and non-cancerous tissues is displayed in Figure 3. The ratio of PART-1 versus actin was used to normalize the data. The difference between PART-1/actin levels in cancerous versus non-cancerous tissues ranged from approximately 0.03 to 0.24.

Our quantitative results were further correlated with qualitative data which were generated by running the amplified samples on gels. For 20 out of the 27 pairs (74%) the data were concordant by both methods (Figure 4). The discordant samples included those with relatively small differences in PART-1 transcripts between cancer and normal tissues.

### DISCUSSION

Through the use of a cDNA microarray-based approach (Lin et al, 2000), were able to identify PART-1 and characterize it as an androgen-induced gene in prostate adenocarcinoma cells, with an expression restricted to the prostate and salivary glands. They further demonstrated that the cDNA sequence of PART-1 encodes for a 60-amino acid protein (Lin et al, 2000).

Our study extends the data related to PART-1 expression and regulation. By using a more sensitive detection system, RT-PCR, rather than the Northern blot analysis used by Lin et al, we were able to detect and verify that PART-1 is also highly expressed in the placenta and thymus and to a lower extent in other tissues. PART-1 is regulated, in prostate carcinoma cells, by a variety of hormones, although primarily by androgens. It is possible that the up-regulation of PART-1 by hormones other than androgens may by due to cross talk between these hormones and the mutant androgen receptor of LNCaP cells (Veldscholte et al, 1992; McDonald et al, 2000). This mutant androgen receptor loses its binding specificity to androgens.

The finding that PART-1 is frequently present at higher levels in malignant than in benign tissue, suggests that PART-1 expression is

© 2001 Cancer Research Campaign 

**Table 2** RT-PCR analysis of PART-1 transcripts with quantitation by LightCycler technology and by qualitative gel electrophoresis

| Case# | Tissue type | Patient age | PART-1* | Actin* | PART-1/Actin | Change in Cancer vs. Normal ** Quantitative PCR | Qualitative PCR |
|---|---|---|---|---|---|---|---|
| 357 | Normal (N) | 65 | 494 | 21200 | 0.023 | | |
| | Cancer (C) | | 461 | 11840 | 0.038 | UP | UP |
| 381 | N | 61 | 273 | 275200 | 0.001 | | |
| | C | | 1937 | 25970 | 0.074 | UP | UP |
| 403 | N | 58 | 1483 | 62440 | 0.023 | | |
| | C | | 148 | 128000 | 0.001 | DOWN | DOWN |
| 422 | N | 66 | 797 | 113000 | 0.007 | | |
| | C | | 4626 | 28140 | 0.164 | UP | UP |
| 423 | N | 54 | 109 | 29100 | 0.003 | | |
| | C | | 167 | 41450 | 0.004 | EQUAL | EQUAL |
| 443 | N | 66 | 807 | 60900 | 0.013 | | |
| | C | | 2812 | 28540 | 0.098 | UP | EQUAL |
| 464 | N | 64 | 1391 | 42760 | 0.032 | | |
| | C | | 579 | 14300 | 0.040 | UP | DOWN |
| 496 | N | 65 | 180 | 88670 | 0.002 | | |
| | C | | 851 | 22800 | 0.037 | UP | UP |
| 506 | N | 67 | 59 | 219 | 0.272 | | |
| | C | | 679 | 2823 | 0.240 | EQUAL | UP |
| 548 | N | 61 | 38 | 5692 | 0.006 | | |
| | C | | 455 | 6346 | 0.071 | UP | UP |
| 554 | N | 71 | 602 | 11070 | 0.054 | | |
| | C | | 7459 | 12800 | 0.066 | UP | UP |
| 573 | N | 50 | 326 | 89090 | 0.003 | | |
| | C | | 2329 | 69120 | 0.033 | UP | UP |
| 598 | N | 65 | 647 | 76920 | 0.008 | | |
| | C | | 1231 | 5033 | 0.244 | UP | UP |
| 638 | N | 62 | 79 | 121300 | 0.001 | | |
| | C | | 198 | 42700 | 0.004 | UP | EQUAL |
| 667 | N | 67 | 773 | 16020 | 0.048 | | |
| | C | | 747 | 36850 | 0.020 | DOWN | EQUAL |
| 689 | N | 63 | 1156 | 14740 | 0.078 | | |
| | C | | 1040 | 19920 | 0.052 | DOWN | UP |
| 697 | N | 68 | 90 | 1116 | 0.080 | | |
| | C | | 140 | 4054 | 0.034 | DOWN | UP |
| 708 | N | 68 | 844 | 9240 | 0.091 | | |
| | C | | 2074 | 11900 | 0.174 | UP | UP |
| 863 | N | 59 | 1003 | 11750 | 0.085 | | |
| | C | | 62 | 352 | 0.177 | UP | EQUAL |
| 870 | N | 63 | 740 | 35590 | 0.020 | | |
| | C | | 782 | 28530 | 0.027 | UP | UP |
| 905 | N | 66 | 476 | 24110 | 0.019 | | |
| | C | | 2443 | 4045 | 0.603 | UP | UP |
| 1120 | N | 58 | 903 | 12430 | 0.072 | | |
| | C | | 1237 | 4155 | 0.297 | UP | UP |
| 1128 | N | 68 | 202 | 192 | 1.054 | | |
| | C | | 426 | 2804 | 0.152 | DOWN | UP |
| 1136 | N | 62 | 123 | 15860 | 0.007 | | |
| | C | | 665 | 12240 | 0.054 | UP | EQUAL |
| 1137 | N | 69 | 49 | 2629 | 0.018 | | |
| | C | | 442 | 2547 | 0.173 | UP | UP |
| 1080 | N | 65 | 448 | 38120 | 0.011 | | |
| | C | | 67 | 38590 | 0.001 | DOWN | UP |
| 1119 | N | 63 | 193 | 36970 | 0.005 | | |
| | C | | 26 | 21940 | 0.001 | DOWN | UP |

* Arbitrary units. **UP = increase in PART-1 expression compared to normal tissue; DOWN = decrease in PART-1 expression compared to normal tissue; EQUAL = equal PART-1 expression in both tissue.

altered during cancer and that its regulation follows different mechanisms than PSA, which is usually found in higher levels in benign tissue (Magklara et al, 2000). The data with PSA, which were derived by using tissue extracts from the same patients, further confirm that PART-1 overexpression is not due to higher levels of luminal epithelium in the cancerous tissues, since PSA is also produced by prostatic luminal epithelium. PART-1 may be a secreted protein and its overexpression in cancer lead us to hypothesize that PART-1 may have value as an additional circulating biomarker for prostate cancer. This possibility merits investigation.

PART-1 is localized to human chromosome 5q12 and encodes for a 60-amino acid protein (Lin et al, 2000). The small size of this

*© 2001 Cancer Research Campaign*

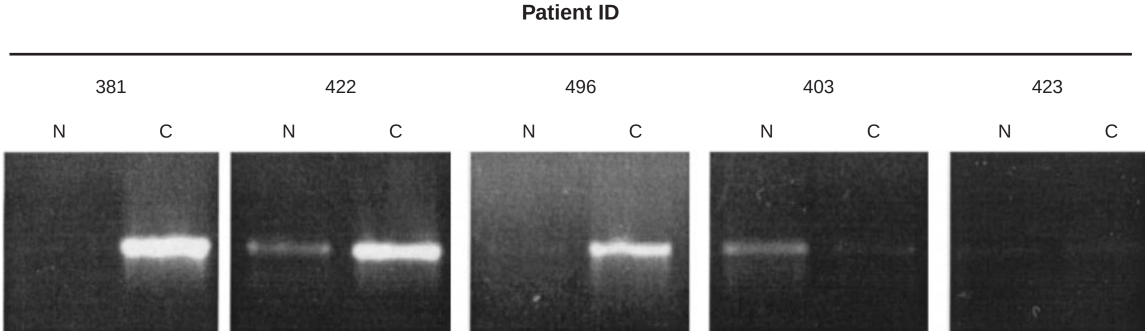

**Figure 4** Selected paired samples of normal (N) and cancerous (C) tissues amplified for PART-1 gene expression. Patients 381, 422, and 496 display an increase in PART-1 expression in the cancerous tissue (see also Figure 3 for quantitative data). Patient 403 has more PART-1 transcript in the normal tissue while patient 423 depicts equal PART-1 expression in both tissues polypeptide, along with its hormonal regulation and its restricted tissue expression pattern, indicates that PART-1 has characteristics reminiscent of a hormone or a growth factor. Isolation of the protein and detailed functional and homology analysis should further illuminate PART-1's function.

In conclusion, our finding of overexpression of PART-1 in a significant percentage of prostate cancers, provides a foundation for future studies examining the potential of PART-1 as a prostate cancer marker and its biological function in the prostate and other tissues.

### ACKNOWLEDGEMENTS

We would like to thank the Natural Sciences and Engineering Research Council of Canada (NSERC) for providing a summer studentship to one of the authors (MS).

### REFERENCES

Black MH and Diamandis EP (2000) The diagnostic and prognostic utility of prostate-specific antigen for diseases of the breast. *Breast Cancer* **59**: 1–14

Diamandis EP (2000) Prostate-specific antigen: a cancer fighter and a valuable messenger? *Clin Chem* **46**: 896–900

Heese A, Becker C, Noldus J, Graefen M, Huland E, Huland H and Lilja H (2000) Human glandular kallikreins 2: a potential serum marker for predicting the organ confined versus non-organ confined growth of prostate cancer. *J Urol* **163**: 1491–1497

Lin B, White JT, Ferguson C, Bumgarner R, Friedman C, Trask B, Ellis W, Lange P, Hood L and Nelson PS (2000) PART-1: A novel human prostate-specific, androgen-regulated gene that maps to chromosome 5q12. *Cancer Res* **60**: 858–863

Magklara A, Scorilas A, Catalona WJ and Diamandis EP (1999) The combination of human glandular kallikrein and free prostate-specific antigen (PSA) enhances discrimination between prostate cancer and benign prostatic hyperplasia in patients with moderately increased total PSA. *Clin Chem* **45**: 1960–1966

Magklara A, Scorilas A, Stephan C, Kristiansen GO, Hauptmann S, Jung J and Diamandis EP (2000) Decreased concentrations of prostate-specific antigen and human glandular kallikrein 2 in malignant versus nonmalignant prostatic tissue. *Urology* **56**: 527–532

McCormack R, Rittenhouse HG, Finlay JA, Sokoloff RL, Wang TJ, Wolfert RL, Lilja H and Oesterling E (1995) Molecular forms of prostate-specific antigen and the human kallikrein gene family: A new era. *J Urol* **45**: 729–744

McDavid K, Melnik TA and Derderian H (2000) Prostate cancer screening trends of New York State men at least 50 years of age, 1994 to 1997. *Prev Med* **31**: 195–202

McDonald S, Brive L, Agus DB, Scher HI and Ely KR (2000) Ligand responsiveness in human prostate cancer: structural analysis of mutant androgen receptors from LNCaP and CWR22 tumors. **60**: 2317–2322

Moran WP, Cohen SJ, Preisser JS, Wofford JL, Shelton BJ and McClatchey MW (2000) Factors influencing use of the prostate-specific antigen screening test in primary care. *Am J Manag Care* **6**: 315–324

Oesterling JA (1991) Prostate-specific antigen: a critical assessment of the most useful tumor marker for adenocarcinoma of the prostate. *J Urol* **145**: 907–923

Rhim JS (2000) Molecular and genetic mechanisms of prostate cancer. *Radiat Res* **155**: 128–132

Rittenhouse HG, Finlay JA, Mikolajczyk and Partin AW (1998) Human kallikrein 2 (hK2) and prostate-specific antigen (PSA): Two closely related, but distinct, kallikreins in the prostate. *Crit Rev Clin Lab Sc*. **35**: 275–368

Veldscholte J, Berrevoets CA, Ris-Stalpers C, Kuiper GG, Jenster G, Trapman J, Brinkmann AO and Mulder E (1992) The androgen receptor in LNCaP cell lines contains a mutation in the ligand binding domain which affects steroid binding characteristics and response to antiandrogens. *J Steroid Biochem Mol Biol* **41**: 665–669

Webb V and Holmes A (2000) Urological cancers: do early detection strategies exist? *BJU Int* **86**: 996–1000

© 2001 Cancer Research Campaign
